# Blunt injury to the inferior gluteal artery: case report of a rare "near miss" event

**DOI:** 10.1186/1754-9493-2-27

**Published:** 2008-10-29

**Authors:** Qi Zhang, Huaijun Liu, Wade R Smith, Jinshe Pan, Wei Chen, Yingze Zhang

**Affiliations:** 1 Department of Orthopaedics, 3rd Hospital, Hebei Medical University, Shijiazhuang, Hebei 050051, PR China; 2 Department of Radiology, 2ed Hospital, Hebei Medical University, Shijiazhuang, Hebei 050051, PR China; 3 Department of Orthopaedics, Denver Health Medical Center, University of Colorado School of Medicine, Denver, CO 80204, USA

## Abstract

Traumatic injuries of the inferior gluteal artery are rare, the majority of which are aneurysms due to sharp or blunt trauma. We report the rare case of a "near miss" event of a patient with an acute hemorrhagic mass in the right buttock caused by blunt trauma to the inferior gluteal artery without "hard" clinical signs of vascular injury. Despite the unusual presentation, diffuse injury of the inferior gluteal artery branches was diagnosed by ultrasonography and angiography. This article highlights the importance of considering an arterial injury following blunt trauma to the buttock with subsequent pain and swelling. Appreciation of this rare injury pattern is necessary in order to facilitate rapid diagnosis and appropriate treatment.

## Background

Gluteal artery injury is uncommon and the injury of the inferior branch (IGA) is more rare than that of the superior branch (SGA)[1]. In the past 30 years, 21 cases of traumatic IGA injury have been reported in the world literature [1-12]. There were 16 cases due to sharp injury, 5 cases due to blunt injury including 4 cases falling onto the buttock. All patients survived the injuries including one case suffering massive blood loss due to delayed diagnosis and treatment[11]. To our knowledge, diffuse breakage of the IGA branches due to falling onto the buttock has not been reported previously. The present case report illustrates the need to consider the diagnosis of arterial injury, and the definitive role of timely angiography in diagnosis and treatment.

## Case presentation

A 63-year-old man presented to the emergency department with a painful swelling in the right buttock. Three hours prior, the man was walking along the road, tripped and fell down with his right buttock directly impacting the edge of the road. The patient felt moderate pain in the right buttock after the injury. He continued walking. Thirty minutes later he began to suffer increasing pain and pressure in the right gluteal area, with increased swelling, firmness and warmth. Upon presentation in our Emergency department 3 hours after the initial injury, the patient developed pain and a large mass in the right buttock with tingling radiating down his right leg. The symptoms aggravated with straightening of the right leg. The patient denied fever, chills, dizziness, nausea, vomiting or shortness of breath. The patient had been undergoing antiplatelet therapy with traditional Chinese medicine for 1 year due to a previous cerebral infarction.

**Figure 1:**
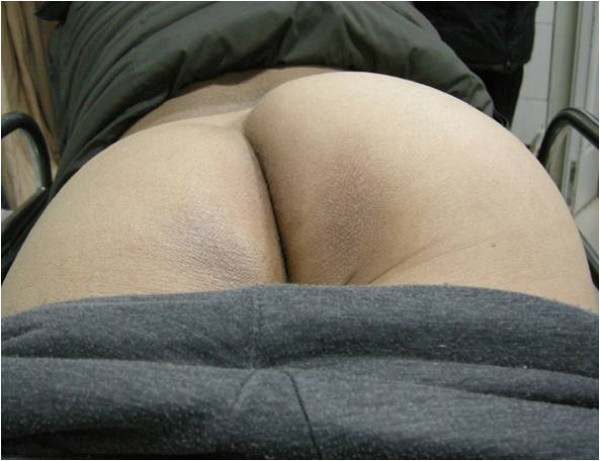
**The patient lies prone, the right gluteal region is obviously more swollen than the left.** The swelling measures about 15 cm × 8 cm from the superior medial to the inferior lateral gluteal region.

On clinical examination, vital signs were normal except blood pressure of 150/110 mm Hg and complaint of 10/10 pain. There was a firm swelling 15 cm × 8 cm in the right gluteal region, from the superior medial gluteal region to the inferior lateral gluteal region (Figure 1). The swelling was tender and warm on palpation but not pulsatile. No bruit was appreciated on auscultation. Bilateral symmetrical pedal pulses were present. Neurological examination revealed the stolidity of the sciatic nerve.

**Figure 2:**
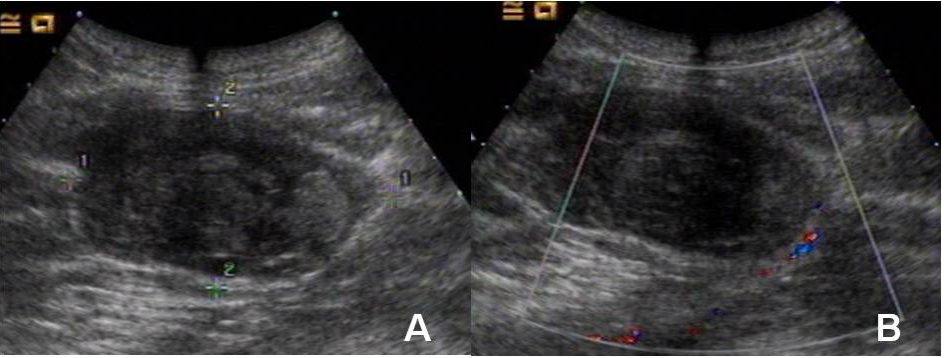
**An ultrasonographic examination of the mass demonstrated lobulated low level densities in the gluteus maximus muscle (panel A)**. A color-flow Doppler ultrasonography was performed without abnormal color signal within the hemorrhagic mass (panel B).

**Figure 3:**
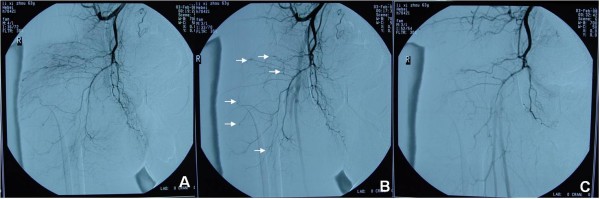
**The arteriogram reveals a diffuse leakage of contrast medium.** Arrows represent ruptured branches of inferior gluteal artery (panels A, B). Arteriography after interventional embolization show no leakage of contrast medium (panel C).

The provisional diagnosis was injury of the SGA with hematoma and pressure on the sciatic nerve. X-ray showed no fracture in the right hip or pelvis. An ultrasonographic examination of the mass demonstrated lobulated low densities in the gluteus maximus muscle implying multiple fluid collections in the muscular layers (Figure 2, panel A). A color-flow doppler ultrasonography was performed without abnormal color signal and evidence of a bleeding stream within the hemorrhagic mass (Figure 2, panel B). The patient then urgently underwent an arterial catheterization and pelvic angiogram in the interventional radiology suite. Selective angiography was performed. The right common iliac artery, right internal iliac artery and its branches were checked separately. The arteriography showed normal flow of the SGA and diffuse leak of contrast medium in the branches of the right IGA (Figure 3, panels A, B). The diagnosis was revised to diffuse breakage of the IGA branches. Gelatin sponge was infused into the right IGA and no leak of contrast medium was found in the follow-up angiogram (Figure 3, panel C). The patient's postoperative course was uneventful and he was discharged three days after the procedure. He went on to an uneventful recovery with resumption of normal work and activity levels.

## Discussion

The main arteries to the gluteal region are the IGA and SGA. It was once reported that the SGA supplied blood to the superior half of the gluteus maximus and the IGA supplied blood to the inferior half of the muscle[13]. However, Song *et al*. demonstrated that the dominant IGA pattern showed higher frequency than the dominant SGA pattern among the four gluteal artery patterns and the branches of IGA distributed blood to a larger area than did those of the SGA[14]. The course of the muscular branches of the SGA and the IGA are in the lateral or inferolateral direction. The SGA perforators were found adjacent to the medial two-thirds of a line drawn from posterior inferior iliac spine to greater trochanter of the femur and the IGA perforators were concentrated along a line in the middle third of the gluteal region above the gluteal crease[15]. The course of IGA perforating vessels is more oblique through the substance of gluteus maximus muscle than the course of SGA perforators[14]. When falling down onto the ground, the IGA and its branches are anatomically more at risk than the SGA.

Why minor falls may cause major arterial injury to the IGA is unknown. Likely there are specific patient predilections such as low compliance of arteries or anticoagulation. The patient in this case had suffered from cerebral thrombosis one year previously which maybe imply intrinsic arterial disease. He was also taking an antiplatelet traditional Chinese medicine. These treatments may affect the normal vascular coagulation function. Kuzuya *et al*. reported a similar case of a patient suffering an IGA bleed without history of trauma whose only risk factor was antiplatelet therapy[16].

The direction and position of the hemorrhagic mass on clinical examinination is not pathognomonic for the specific arterial injury. Diagnostic techniques for arterial lesions include ultrasonography, color-flow doppler ultrasonography, aspiration, computed tomography, magnetic resonance imaging and angiography[4]. In this case, the injury of the IGA could not be distinguished clinically from that of SGA. Ultrasonography was chosen as primary test which demonstrated lobulated low-density signals. These findings led to the more definitive test for arterial bleeding and intervention: angiography. Angiography is the major method to diagnose arterial injury with several benefits including the ability to identify the distal and proximal tributaries of the affected artery and the ability to combine diagnosis and treatment during a single examination[3]. Helical CT or CT-angiogram can also be applied to determine vascular hemorrhage[6]. If considered as high flow hemorrhage, vascular injuries must be treated urgently.

Arterial lesions may be repaired using either surgical or less invasive techniques, such as embolization during angiography. However, in this case, surgery would likely have caused more injury to the IGA branches and have required an extensive open wound. Keeling *et al*. reported a patient with a traumatic IGA pseudoanuerysm who suffered life-threatening blood loss due to delayed diagnosis and treatment[11]. The patient was intially treated by surgical exploration. Three days later, there was a large, acute bleed from the previous surgical wound site. Despite repeat packing, the blood loss failed to cease following 10 units of packed red blood cells. However, selective angiographic embolization of such cases is an effective and reliable method to stop arterial bleeding, especially in the pelvic region, with minimal invasion and improved outcomes[17]. The advantages of angiography with embolization include a decreased risk of infection, the avoidance of opening the retroperitoneal space and decreased risk of iatrogenic nerve and arterial injuries[1]. In the present case report, embolization was performed urgently to decrease bleeding and avoid permanent damage to the sciatic nerve from the expanding hematoma.

## Conclusion

The differential diagnosis of any acutely expanding gluteal mass following blunt trauma should include an arterial injury. Immediate angiography should be considered as a diagnostic and therapeutic tool.

## Competing interests

The authors declare that they have no competing interests.

## Authors' contributions

All authors contributed equally to this case report. All authors read and approved the final version of the manuscript.
